# Detection of amitraz resistance and reduced treatment efficacy in the Varroa Mite, *Varroa destructor*, within commercial beekeeping operations

**DOI:** 10.1371/journal.pone.0227264

**Published:** 2020-01-17

**Authors:** Frank D. Rinkevich

**Affiliations:** USDA-ARS Honey Bee Breeding, Genetics, and Physiology Laboratory, Baton Rouge, Louisiana, United States of America; University of Crete, GREECE

## Abstract

The parasitic mite *Varroa destructor* and the associated viruses it transmits are responsible for most instances of honey bee colony losses in the United States. As such, beekeepers utilize miticides to control Varroa populations. Widespread resistance has developed to the miticides fluvalinate and coumaphos. However, Varroa has largely maintained susceptibility to amitraz despite a long and extensive use history. Anecdotal reports of reduced amitraz effectiveness have been a widely discussed contemporary issue among commercial beekeepers. Amitraz resistance was measured by *in vitro* bioassays with technical amitraz as well as Apivar^®^ efficacy tests. Amitraz resistance was evaluated in commercial beekeeping operations in Louisiana, New York, and South Dakota with a long history of amitraz use. This research shows that amitraz remains an effective Varroa control product in many operations. However, apiaries across operations displayed a wide range of amitraz resistance from no resistance to high resistance that resulted in Varroa control failure. The resistance ratios from *in vitro* amitraz bioassays were correlated with reduced Apivar^®^ efficacy, demonstrating *bona fide* cases of Varroa control failures due to amitraz resistance. Therefore, amitraz resistance monitoring protocols need to be developed. A resistance monitoring network should be established to ensure the sustainability of miticide use for Varroa control.

## Introduction

Pesticide resistance is a phenomenon by which organisms can survive higher doses or concentrations of a toxic substance that previously resulted high levels of mortality. Resistance to many classes and types of pesticides occurs in many arthropod species [1, 2]. Resistance may develop extremely rapidly [3, 4], occur over a broad geographic area [5–7], evolve in multiple and independent instances [8–10], and resistance levels may be extremely high [11–13]. Major mechanisms of resistance include enhanced detoxification [14–17], target-site insensitivity [18–20], and reduced cuticular penetration [21].

The parasitic mite, *Varroa destructor* (hereto referred to as Varroa), is the most critical cause of honey bee colony loses in commercial beekeeping operations [22–25]. Since its arrival in the late 1980s, the detrimental impact of Varroa has amplified in commercial beekeeping operations in the USA [26]. Varroa feeds destructively on fat body in the pupae and adults [27] and also transmits many viruses. Varroa-transmitted viruses such as deformed wing virus (DWV) and black queen cell virus (BQCV) are closely associated with high colony loses [24, 26, 28].

Due to the critical threat from Varroa, commercial beekeepers have employed a number of synthetic miticides for Varroa control. Resistance to many of these materials has developed [29]. Products containing tau-fluvalinate (a pyrethroid that targets the voltage-gated sodium channel [30]) or coumaphos (an organophosphate that targets acetylcholinesterase [31]) have been used to control Varroa early in the invasion in the USA. However, excessive and exclusive use of these materials resulted in high levels of resistance and widespread control failure [29, 32, 33]. It is possible that resistance developed because they persist at high concentrations in the wax and represent a constant exposure [34, 35]. Amitraz (a formamidine that targets octopamine/tyramine receptors [36]) has been used to control Varroa populations for more than 20 years in the USA. Although amitraz resistance was initially reported shortly thereafter its initial use [37], subsequent outbreaks of amitraz resistance have not been documented. However, contemporary anecdotes suggest reduced efficacy and highly variable success of amitraz in commercial beekeeping operations. Despite a plethora of circumstantial suggestions, the mostly likely explanation of reduced Varroa control via amitraz indicates amitraz-resistant Varroa populations. Amitraz resistance in Varroa is plausible because isolated incidents of amitraz resistance were reported in the USA [37], Argentina [38], and Czechia [39]. However, widespread amitraz resistance in Varroa in commercial beekeeping operations has not been documented in the USA.

The goals of this research are to 1) establish baseline data on an amitraz-sensitive Varroa population, 2) identify the prevalence and intensity of amitraz resistance in commercial beekeeping operations with a history of amitraz use, and 3) verify that reduced Apivar^®^ efficacy is correlated with amitraz resistance. The current predictions are 1) commercial beekeeping operations with a long history of high level amitraz use exhibit amitraz-resistant Varroa populations, 2) amitraz resistance is consistent within apiaries within an operation, and 3) reduced Apivar^®^ efficacy correlates with increased amitraz resistance.

## Materials and methods

### Apiary locations

Baseline amitraz toxicity was determined from Varroa in colonies at the USDA-ARS Honey Bee Breeding, Genetics, and Physiology Laboratory in Baton Rouge, LA. These Varroa were used as an isolated amitraz-susceptible reference because the colonies are maintained without amitraz application and very few colonies are imported.

Varroa were collected from commercial beekeeping operations in Louisiana, New York, and South Dakota that have had a >3-year history of amitraz use for Varroa control. Apiaries in Louisiana were sampled in April 2019. Apiaries in New York and South Dakota were sampled in July and August 2019.

### Varroa collection

Varroa were harvested using the powdered sugar drop method adapted for large scale collection [40]. Varroa were collected from 6 to 8 colonies in each apiary (representing 10–15% of the colonies in the apiary) by shaking bees from 3 combs containing open and sealed brood into a 19 L plastic bucket with approximately 100 mL of powdered sugar. The bucket was covered with a lid and gently inverted constantly for 1 minute to dislodge Varroa. The contents of the bucket were poured in to a 0.6 cm mesh hardware cloth screen that was fit snugly within another 19 L bucket. The bucket was covered with a lid and Varroa were allowed to drop for 5 minutes. The powdered sugar and Varroa at the bottom of the bucket were poured in to a fine mesh strainer. The Varroa were consolidated in to a holding bucket in the field. Varroa were sorted for bioassays by collecting mites and powdered sugar in to a fine mesh strainer and rinsed with water. Varroa were emptied on to a paper towel on a plastic tray. Live Varroa were transferred to treated vials using a fine haired paint brush. Amitraz resistance and Apivar^®^ efficacy were not able to be evaluated in apiaries in which fewer than 20 total Varroa mites were collected with this method.

### Amitraz bioassays

Stock solutions of technical grade amitraz (>97% purity, ChemService, West Chester PA) were prepared in acetone. Vial bioassays followed previously reported methods with some modifications [37, 41]. A 0.5 mL volume of amitraz stock solution was added to a 20 mL scintillation vial. Control vials were treated with acetone. Vials were placed on their side and rolled until acetone was evaporated. Treated vials were dried for at least an additional hour.

A white-eyed honey bee pupa that was not infested with Varroa was added to the vial. A total of 10 Varroa collected as described above were transferred in to vials, and vials were sealed with a piece of parafilm. Holes were poked through the parafilm to allow ventilation. Vials were laid on their side in a plastic tray, secured with rubber bands, and kept in an incubator at 33 ± 1°C with >50% RH in continuous darkness. Mortality was recorded 24 hours after treatment. The LC_50_ value was calculated by probit analysis with Abbott’s correction for control mortality [42] using Minitab (State College, PA). The LC_50_ values were significantly different if the 95% CI did not overlap between populations. Resistance Ratios (RR) were calculated relative to the amitraz LC_50_ of the amitraz-sensitive USDA Lab population. The correlation of amitraz LC_50_ with the concentration-response curve slope was analyzed by using Spearman Rank Correlation (JMP 12.2). The amitraz bioassay was not performed in apiary D4 due to low Varroa collection.

### Apivar^®^ efficacy

**Fig 1:**
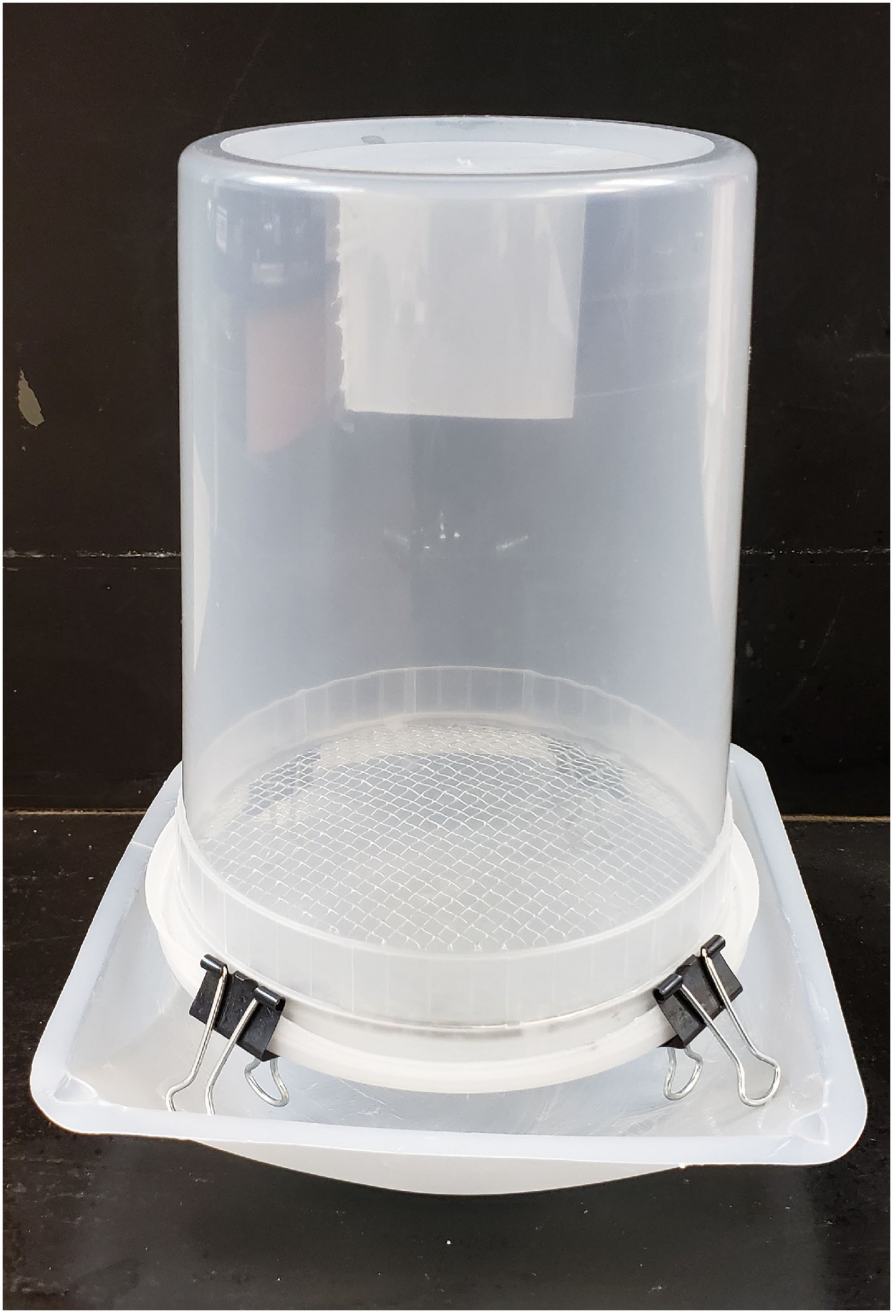
Cage design for Apivar^®^ efficacy test. A square of Apivar^®^ was hot glued to the bottom of a plastic container. The screen lid was used to seal the container after bees were added. The container was inverted and suspended over a weighing dish covered in petroleum jelly using binder clips.

Apivar^®^ efficacy was evaluated based on modifications of previously published methods [32, 43]. A 4 cm x 4 cm square was cut from an Apivar^®^ strip. This square was hot glued perpendicularly to the bottom of a disposable 946 mL polypropylene container. These assay cups were constructed the morning they were to be used and no cups were reused. Adult bees from 2 brood frames were shaken into a 19 L plastic bucket. A 120 mL plastic cup was used to scoop approximately 300 bees into the container with an Apivar^®^ square. A lid with 0.3 cm mesh hardware cloth was used to close the container. Four binder clips were attached to the lid and the latches folded backwards. The cup was inverted over a plastic weighing dish coated with a thick layer of petroleum jelly (Fig 1). Control cups without an Apivar^®^ square was used to evaluate background Varroa drop. Bees were held in the shade at ambient field conditions and Varroa were allowed to drop on to the weighing dish. The shortest time interval for 100% Apivar^®^ efficacy in the amitraz sensitive USDA Lab population was determined by recording the number of mites that dropped every 30 minutes for up to 6 hours. Varroa were exposed to the Apivar^®^ strip in all the other apiaries for the time it took for 100% Apivar^®^ efficacy in the USDA Lab population. Bees were then washed in warm soapy water as previously described to dislodge any remaining Varroa [44]. The number of remaining Varroa was added to the number of dropped Varroa to determine the number of total Varroa in the bee sample. The number of dropped Varroa was divided by total Varroa to calculate Apivar^®^ efficacy. The total number of Varroa was divided by the number of bees to determine the Varroa infestation rate. The Apivar^®^ efficacy test was not performed in apiaries A1, D1, E, and F because Apivar^®^ efficacy test was not developed at that time.

The following statistics were performed using JMP 12.2. Apivar^®^ efficacy was compared using Kruskal-Wallis Test. Variation in Apivar^®^ efficacy was compared using Welch’s Test for Unequal Variance. The correlation of amitraz resistance ratios and Apivar^®^ efficacy was compared using Spearman Rank Correlation.

## Results

Amitraz resistance in vial bioassays or Apivar^®^ efficacy tests were not able to be evaluated in 5 of 11 commercial beekeeping operations (45%) and 7 of 19 apiaries (37%) due to low mite collection in the sugar drop method.

The results of the amitraz bioassays are shown in Table 1. The USDA-Lab population had an amitraz LC_50_ of 0.008 μg/vial and this was the lowest LC_50_ value reported. A range of amitraz resistance ratios was found in commercial beekeeping operations with apiaries showing no resistance (LC_50_ not significantly different from the USDA Lab population), low resistance (<5-fold RR), medium resistance (5- to 10-fold RR), and high resistance (>10-fold RR). Apiaries with high amitraz resistance (>10-fold RR) were considered control failures because high Varroa infestation was observed despite an active amitraz application. There was a significant negative correlation between the amitraz LC_50_ and slope of the concentration-response curve (Spearman Rank Correlation: ρ = -0.6414, p = 0.0246).

**Table 1:** Amitraz vial bioassay results. The LC_50_ is in units of μg/vial. Letters in the Apiary column indicate a commercial beekeeping operation and numbers indicate different apiaries within that operation. LC_50_ values with the same superscript letters are statistically similar.

Apiary	N	LC_50_ (95% CI)	Slope (SE)	RR
USDA-Lab	201	0.008 (0.006–0.009) ^A^	3.9 (0.6)	1.0
A1	201	0.014 (0.010–0.017) ^BJ^	2.2 (0.3)	1.7
A2	118	0.031 (0.021–0.045) ^CDEHIJ^	1.8 (0.4)	3.9
A3	126	0.053 (0.037–0.077) ^CDGH^	3.1 (1.1)	6.6
A4	290	0.021 (0.017–0.025) ^CDEIJ^	2.3 (0.3)	2.6
B	119	0.180 (0.082–0.394) ^F^	0.8 (0.4)	22.5
C	118	0.076 (0.042–0.138) ^CDFGH^	1.5 (0.4)	9.5
D1	382	0.106 (0.085–0.132) ^F^	1.4 (0.2)	13.2
D2	185	0.063 (0.049–0.080) ^DGH^	2.2 (0.4)	7.9
D3	143	0.050 (0.036–0.066) ^CDGH^	2.0 (0.5)	6.2
E	144	0.026 (0.021–0.033) ^CDIJ^	2.2 (0.4)	3.2
F	243	0.014 (0.007–0.025) ^ABCDIJ^	1.8 (0.4)	1.7

**Fig 2:**
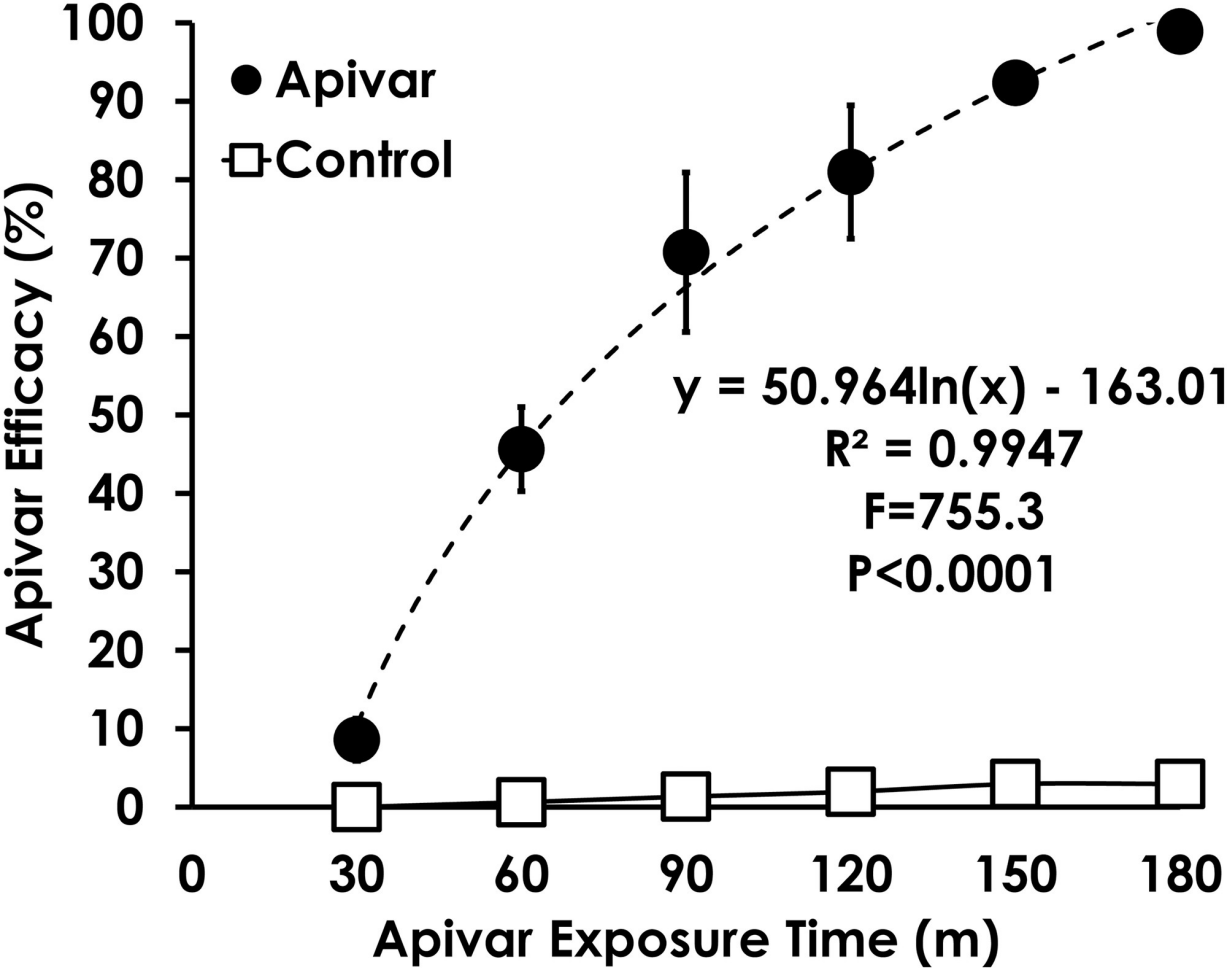
Time course of Apivar^®^ efficacy in amitraz-susceptible Varroa. Apivar^®^ was 100% effective at removing Varroa in the amitraz-susceptible population at 3 hours.

Apivar^®^ efficacy was 100% after 3 hours in the amitraz-susceptible USDA Lab population (Fig 2). The time course of Apivar^®^ efficacy was best fit with a 1-parameter logistic function. Varroa drop was mostly due to Apivar^®^ exposure because less than 3% of Varroa dropped in the control. Therefore, a 3-hour exposure was used to assess differences in Apivar^®^ efficacy in commercial beekeeping operations.

**Fig 3:**
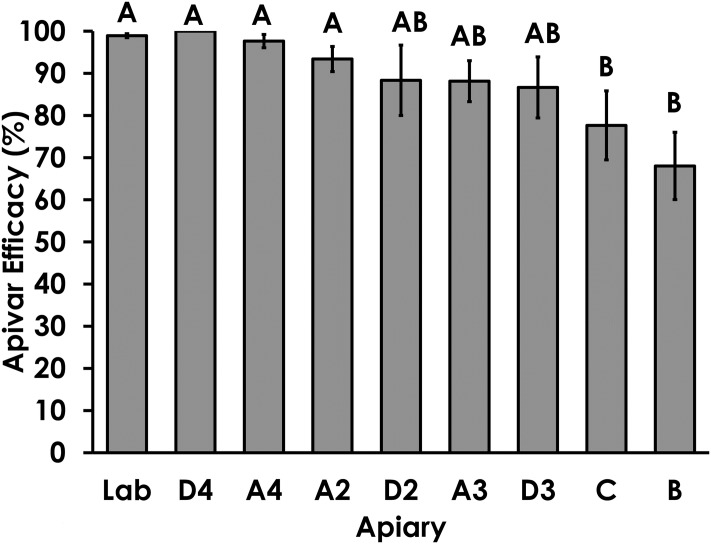
Apivar^®^ efficacy in commercial beekeeping apiaries. Columns with different letters indicate significant differences.

**Fig 4:**
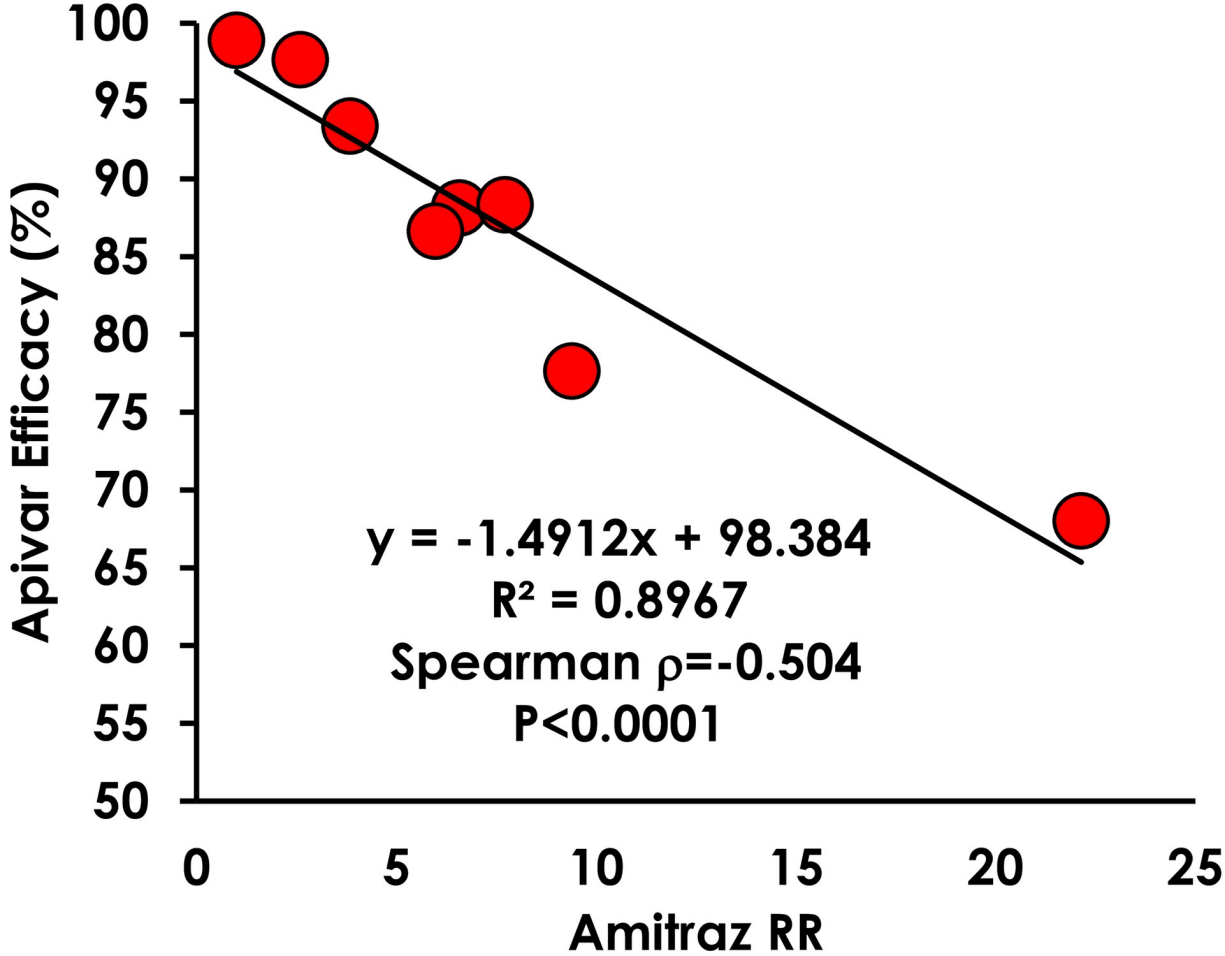
Reduced Apivar^®^ efficacy is correlated with amitraz resistance.

Apivar^®^ efficacy among commercial beekeeping apiaries showed a range of responses from highly effective (>97%), mostly effective (90–97%), somewhat effective (80–90%), minimally effective (<80%; Fig 3). Apivar^®^ efficacy was significantly lower in apiaries B and C compared to the susceptible Lab population (Kruskall-Wallis, χ^22^ = 19.60, df = 7, p = 0.0077). Apivar^®^ efficacy was much more variable among colonies within apiaries with reduced efficacy (Welch’s Test: F = 4.00, DF_Num_ = 7, DF_Den_ = 19.59, p = 0.007). For example, Apivar^®^ efficacy within apiary B was 68%, but ranged from 28% to 97% among colonies. Apivar^®^ efficacy was significantly correlated with amitraz resistance ratios (Spearman Rank Correlation: ρ = -0.504, p<0.0001, Fig 4). Apivar^®^ efficacy was not affected by the number of bees in the test (Spearman Rank Correlation: ρ = -0.2255, p = 0.078), number of Varroa in the test (Spearman Rank Correlation: ρ = -0.1061, p = 0.4116), or Varroa infestation rate (Spearman Rank Correlation: ρ = -0.0459, p = 0.7231).

## Discussion

Ideal conditions exist for the development of amitraz resistance because Varroa has a rapid generation time and capable of producing many offspring while exposed to frequent applications of high concentration amitraz [26, 45]. However, Varroa control failures due to amitraz resistance continue to be rare despite the first reports of amitraz resistance nearly 20 years ago [37]. The fact that nearly half of the commercial beekeeping operations that have relied on amitraz for Varroa control for at least 3 years did not yield enough Varroa to test for amitraz resistance is evidence of the ongoing effectiveness of this treatment. The isolated reports of Varroa control failure due to amitraz resistance [37–39] despite a long history of intense and frequent amitraz use is peculiar as there are numerous widespread reports of Varroa control failure due to high levels of resistance to fluvalinate [17, 32, 46, 47] and coumaphos [33, 48–50].

The low amitraz LC_50_ in the USDA lab population validates it use as an amitraz-susceptible reference population. Establishing an accurate LC_50_ is critical because the data above suggests that an amitraz resistance ratio of >10-fold may lead to control failure. Determining the LC_50_ values to fluvalinate and coumaphos will establish the overall miticide susceptibility and make it a valuable reference Varroa population.

The details of the LC_50_ calculations are important for contextual interpretation. For example, the slope of the concentration-response curves indicate the potential for high levels of resistance because the slope is reduced in resistant populations (Table 1, [51, 52]). The consequence of reduced slopes is that the resistance ratios are much more dramatic if calculated comparing the LC_90_. For example, the amitraz resistance ratios in apiaries B and D1 are 22.1 and 13.0, respectively, when comparing the LC_50_. The amitraz resistance ratios increase dramatically to 374.4 for apiary B and 48.5 for apiary D1 when comparing the LC_90_. This is an important consideration because using discriminating concentration bioassays to detect resistance is built on the assumption that the slopes of the concentration-response curves are equal between populations [52]. Therefore, generating concentration-response curves is the most rigorous and valid method to compare amitraz resistance among Varroa populations.

Amitraz resistance monitoring is complicated by the high inter-colony variation in apiaries with significantly reduced Apivar^®^ efficacy (Fig 3). This colony level resolution suggests that each colony may act an island of resistance with its own distinct Varroa population. Beekeepers have reported inconsistency in amitraz treatment efficacy among colonies within an apiary and this variation seems to support those anecdotal observations.

The results of the Apivar^®^ efficacy test indicate that a small change in Apivar efficacy may be indicative of control failure. The Apivar^®^ efficacy was 68% in apiary B. This apiary was experiencing a Varroa control failure because of high Varroa infestation levels (i.e. 11.45 Varroa/100 bees) during an active amitraz treatment. This level of efficacy would be not be classified as resistant in the decision scale for determining resistance to Apistan^®^ (i.e. fluvalinate [53]). This difference in threshold for resistance determination may be the consequence of differences in the slope of the concentration-response curves for amitraz and fluvalinate. In this study, the high slope of the amitraz concentration-response curve was 3.94 in the susceptible USDA Lab Varroa population, while the slope of fluvalinate concentration-response curves for fluvalinate-susceptible Varroa is near 1 [39, 41]. This is critical because in concentration-response curves with high slopes, small changes in concentration may cause large changes in survivorship, while large changes in concentration are necessary to induce small changes in survivorship in concentration-response curves with low slopes. Therefore, the threshold to determine amitraz resistance is much lower than for fluvalinate.

A functional definition of resistance is important for consistent and practical interpretation of bioassay results. The vial bioassay revealed many significant differences in the amitraz LC_50_ among the apiaries that were sampled (Table 1), while the Apivar^®^ efficacy test showed that Varroa from only apiaries B and C were significantly different from the USDA Lab with 68% and 77% efficacy, respectively (Fig 3). The amitraz RR in apiaries B and C was 22.5 and 9.5, respectively. Therefore, Varroa populations that have an amitraz RR of >10-fold and Apivar^®^ efficacy of <80% can be classified as functionally resistant to amitraz. Because Apivar^®^ efficacy and amitraz resistance are highly correlated (Fig 4), these methods may be used reliably and interchangeably as measurements of amitraz resistance.

It is critically important to identify the beekeeping management factors and variations in amitraz use that contribute to the development of amitraz resistance in order to extend the effectiveness of amitraz to control Varroa. It does not appear that amitraz use alone can account for amitraz resistance because all these operations had >3 years of amitraz use history. It is likely the variations in amitraz use such as the frequency, intensity, and timing are very important drivers of this phenomenon. This is because amitraz resistance and reduced Apivar^®^ efficacy tended to be similar across apiaries within an operation and restricted between apiaries within the same geographic area. However, those factors cannot be accurately determined from this dataset due to the large variation in beekeeping management factors between commercial beekeepers and our relatively small sample size. Another caveat of this study is that it was conducted under a limited scope of conditions. The dynamics of amitraz resistance in Varroa may change at other critical periods in the annual commercial beekeeping schedule such as after amitraz applications in the fall, drift induced by robbing of dying colonies, or during transportation and consolidation in winter holding yards. Continuing this research to assemble a long-term data set on amitraz resistance under the variable conditions in many commercial beekeeping operations will accurately determine the importance of beekeeping management factors on the prevalence, intensity, and trends of amitraz resistance.

## Supporting information

S1 Table (XLSX)
